# Relevance of Chemokines in Mobilizing γδ T Cells in the Biliary Tract Cancer Microenvironment: Potential for γδ T-Cell–Based Adoptive Cell Therapy

**DOI:** 10.1097/COC.0000000000001306

**Published:** 2026-02-17

**Authors:** Saikat Mandal, Arkadeep Dhali, Manideepa Maji, Guruprasad Aithal

**Affiliations:** * School of Medicine, University of Nottingham; † NIHR Nottingham Biomedical Research Centre, Nottingham University Hospitals NHS Trust and the University of Nottingham, Nottingham; ‡ Faculty of Health, Imperial College, London; § Sheffield Teaching Hospitals NHS Foundation Trust, Sheffield; ∥ Hull York Medical School, University of Hull; ¶ Haematology, Hull University Teaching Hospitals NHS Trust, Hull, United Kingdom

**Keywords:** γδ T-cell, adoptive cell therapy, biliary tract cancer, cholangiocarcinoma, gallbladder cancer, chemokines

## Abstract

**Objective:**

Biliary tract cancer (BTC) has a poor prognosis with limited therapeutic options. γδ T cells represent an MHC-independent immune cell population; however, their therapeutic efficacy in solid tumors is constrained by insufficient tumor infiltration. Chemokine-mediated trafficking is fundamental to T lymphocyte recruitment; however, the chemokine landscape of the BTC tumor microenvironment (TME) remains uncharacterized. Using single-cell RNA sequencing of BTC tissues, we delineated chemokine ligand expression patterns, stratified chemokine producers by lineage, assessed γδ T-cell recruitment mechanisms, and identified chemokine-mediated immune escape.

**Methods:**

We analyzed single-cell RNA sequencing data from 3 independent GEO cohorts (GSE210066, GSE201425, and GSE213452; 19 patients) to comprehensively delineate γδ T-cell mobilization-related chemokine expression across the BTC TME using the Seurat v5.0 pipeline in R.

**Results:**

Analysis identified a multiaxis chemokine profile within the BTC TME. High expression of CCL5, CCL4, and CCL3 established predominant CCR5-mediated recruitment axes supporting Vγ9Vδ2 T-cell infiltration, whereas CCL2 and modest CXCL8 supported CCR2^+^ and CXCR1^+^ Vδ1 T-cell recruitment. Notably, CXCL16 expression supported epithelial γδ T-cell homing through CXCR6. However, critical deficiencies in CXCL9 and CXCL10 suppress the IFN-γ-driven immunity. Paradoxically, chemokine axes supporting γδ T-cell recruitment (CCL2-CCR2, CXCL8-CXCR1, CXCL12-CXCR4) simultaneously recruit immunosuppressive populations, such as myeloid-derived suppressor cells (MDSCs), regulatory T cells (Tregs), and tumor-associated macrophages (TAMs).

**Conclusion:**

Comprehensive single-cell analysis identified selective chemokine recruitment signatures supporting γδ T-cell infiltration but revealed paradoxical corecruitment of immunosuppressive populations. Patient stratification through chemokine profiling, combined with γδ T-cell enrichment and targeted chemokine antagonism, represents a rational therapeutic strategy.

Biliary tract cancer (BTC) is a lethal cancer with rising incidence in the UK (∼3000 cases annually) and poor prognosis, with only 13% 3-year survival.^1^ Surgical resection remains the only curative option; however, up to 65% to 70% of patients present with unresectable disease, and those undergoing surgery face substantial recurrence risk, with 80% relapsing within 3 years.^2–4^ Current approved treatments, including adjuvant capecitabine, cisplatin-gemcitabine, immunotherapy agents such as anti-PD1 (eg, pembrolizumab), and targeted therapies such as pemigatinib and ivosidenib, achieve a complete response in only 2% to 5% of advanced cases.^5,6^ Therefore, there is a critical need for novel therapeutic approaches for advanced biliary tract cancers.

γδ T cells comprise <5% of peripheral blood T-lymphocytes, but possess potent antitumor activity independent of classic major histocompatibility complex (MHC) presentation.^7^ BTC frequently downregulates MHC class I antigens, enabling immune evasion by CD8^+^ T cells.^8,9^ In contrast, γδ T cells exhibit rapid activation at peripheral sites without MHC dependence, rendering them potentially effective in MHC-deficient tumors. The Vδ1 subset of γδ T cells engages NKG2D for tumor surveillance, whereas Vγ9Vδ2 T cells recognize phosphoantigens derived from mevalonate pathway metabolites that accumulate in malignant cells.^10^ The activation of Vγ9Vδ2 T cells depends on butyrophilin (BTN) family molecules, particularly BTN3A1, which is significantly upregulated during BTC development, supporting the therapeutic potential of γδ T-cell–based adoptive immunotherapy.^11^

Despite these favorable immunologic attributes, the therapeutic efficacy of γδ T cells in solid tumors remains limited by their insufficient infiltration into tumor tissues and inadequate local expansion, activation, and tumor evasion. The trafficking of T-lymphocytes into tumors is dependent upon chemokine-chemokine receptor interactions, which establish spatial organization, guide directional migration, and organize immune cell homing to specific tissue compartments.^12–14^ Accordingly, the chemokine profile of a given tumor microenvironment (TME) determines the subset composition and infiltration kinetics of the recruited immune cells. If specific chemokine ligands are absent or cognate chemokine receptors are not expressed on transferred γδ T cells, adoptive transfer fails to achieve adequate tumor infiltration, regardless of the intrinsic effector functions of the cells.

Previous studies have identified chemokine receptor expression in γδ T-cell subsets. Vγ9Vδ2 T cells show high levels of CCR5 (responsive to CCL3, CCL4, and CCL5) and CXCR3 (responsive to CXCL10 and CXCL11), moderate expression of CCR2 (responsive to CCL2), and low expression of CXCR4.^15,16^ Vδ1 T cells express CXCR1 (responsive to CXCL5, CXCL6, and CXCL8) and CCR2, with variable expression of CXCR3 and CXCR4 (responsive to CXCL12).^15–17^ Epithelial-associated γδ T cells, in addition, express CCR6 (responsive to CCL20) and CXCR6 (responsive to CXCL16), which facilitates skin and mucosal homing.^18^ These chemokine-mediated receptor-ligand interactions drive the recruitment of directional T-lymphocytes to tumor tissues. A systematic understanding of chemokine expression patterns within the BTC tumor microenvironment (TME) is essential for optimizing γδ T-cell immunotherapy design and identifying chemokine-mediated immune escape pathways that may constrain clinical responses. Single-cell RNA sequencing profiles from publicly available Gene Expression Omnibus (GEO) cohorts were examined to comprehensively delineate γδ T-cell mobilization-related chemokine ligand expression patterns across the BTC tumor microenvironment, identify and stratify chemokine-producing cellular populations by lineage and phenotype, evaluate the cellular and molecular prerequisites for effective γδ T-cell recruitment through chemokine-mediated trafficking, and identify chemokine-mediated immune escape mechanisms.

## METHODS

Single-cell RNA sequencing (scRNA-seq) data sets for BTC were obtained from 3 independent GEO data sets, GSE210066, GSE201425, and GSE213452,^19–21^ encompassing 156,827 cells derived from 19 patient samples, including intrahepatic cholangiocarcinoma, extrahepatic cholangiocarcinoma, and gallbladder malignancy samples. Quality control filtering excluded cells with fewer than 200 detected genes, abnormal UMI counts, mitochondrial content exceeding 15%, hemoglobin expression >3%, and those flagged by dissociation- or sorting-related markers. Library-size normalization employed a log transformation (10,000 UMIs per cell). To minimize technical confounding, log-normalized expression was adjusted using linear regression to remove the effects of ribosomal- and heat-shock response-associated genes. Highly variable features (n=2000) were selected using a variance-stabilizing transformation. Principal component analysis (PCA) dimensionality reduction was preceded by Harmony batch correction^22^ applied across data sets (Fig. 1), with subsequent UMAP visualization (30 PCs). Unsupervised clustering was performed at resolution 0.9 pre- and post-Harmony correction using the Seurat v5.0 pipeline in R. Cluster-specific markers were determined using the Wilcoxon rank-sum testing (min.pct=0.25, logfc.threshold=0.25) against lineage reference genes.

**FIGURE 1:**
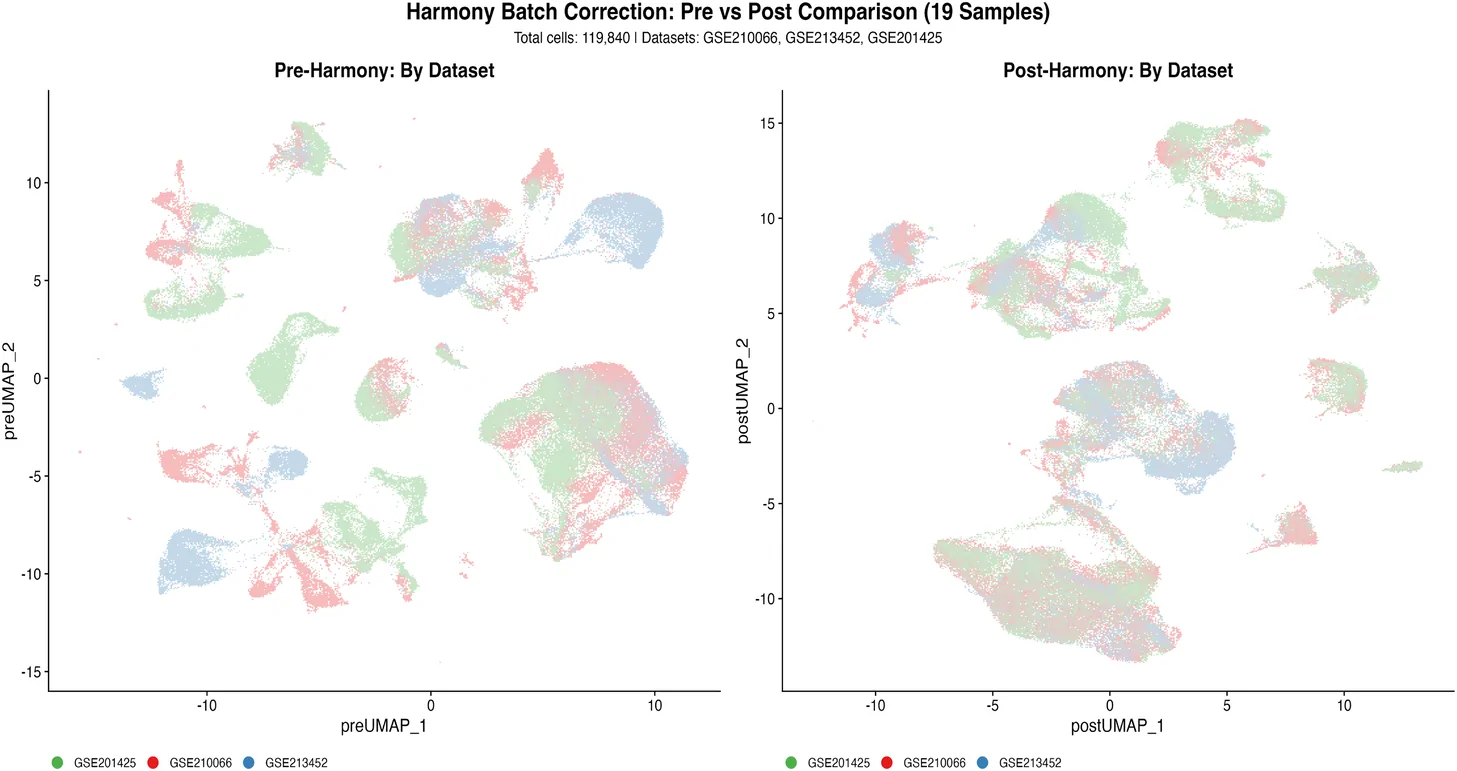
Harmony Batch Correction - Pre vs Post Comparison (19 Samples). Dataset integration visualization. UMAP dimensionality reduction plots comparing harmony batch correction efficacy across three integrated single-cell RNA-sequencing datasets (GSE201425 [green], GSE210066 [red], GSE213452 [blue]). The left panel (Pre-Harmony) shows marked dataset-driven spatial segregation, with each dataset occupying a distinct transcriptomic space, despite representing shared biological populations. The right panel (Post-Harmony) demonstrates successful batch-effect removal, as evidenced by the intermingling of dataset-specific colors and consolidated spatial clustering of biologically equivalent cell populations across all three datasets.

Shared-nearest-neighbor (SNN) graphs were constructed based on Euclidean distances in PCA or Harmony space (*k*=30), with the Louvain algorithm applied to optimize modularity and assign cells to discrete clusters. Cell-type annotation (Fig. 2) was performed using marker gene expression patterns and published reference data sets of immune and epithelial signatures.^23,24^ Cluster stability and composition were evaluated by comparing the cell distribution across data sets before and after harmony correction.

**FIGURE 2:**
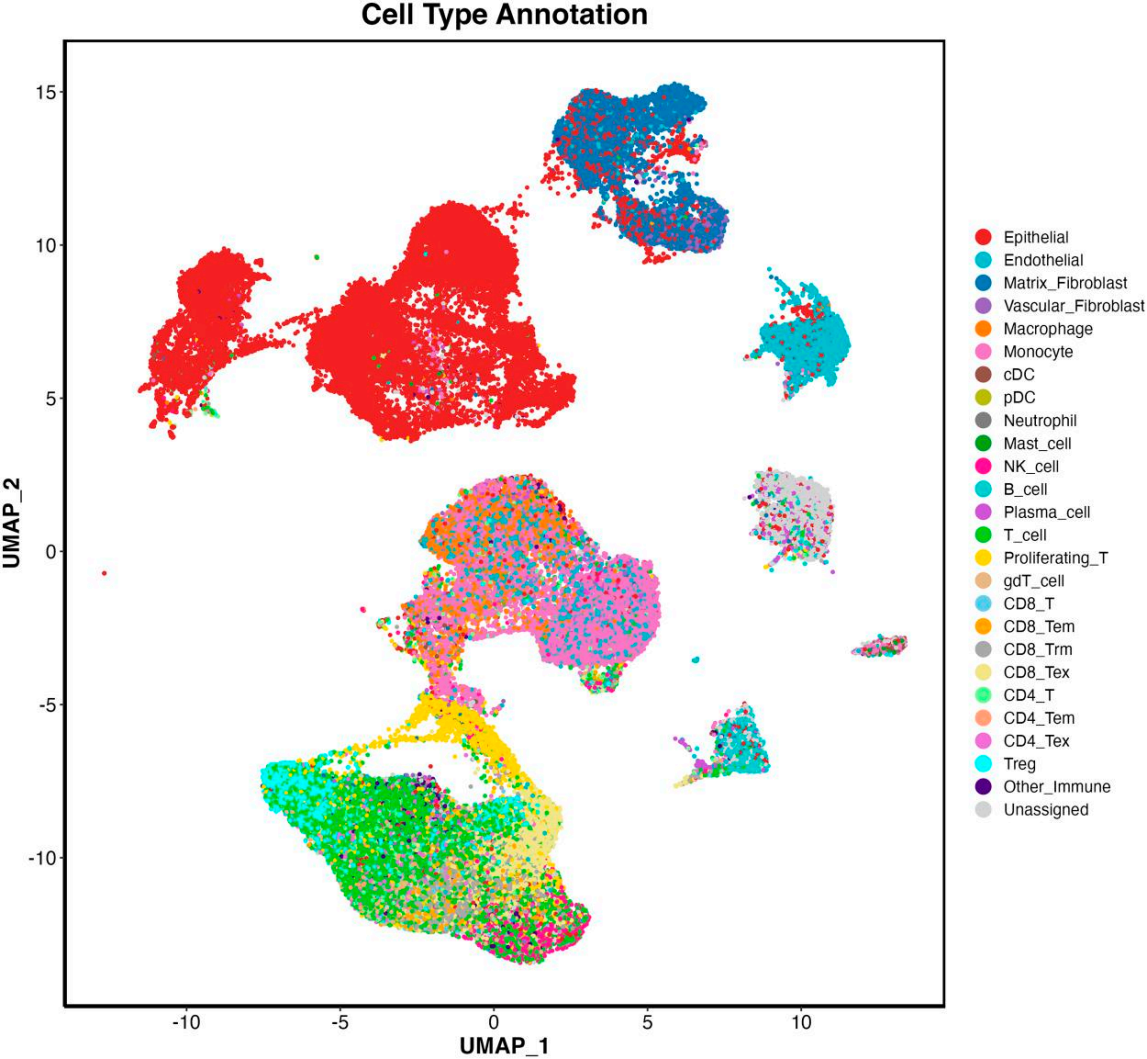
Cell-type annotation of the post-Harmony integrated data set. Cell-type annotation of the integrated BTC microenvironment. The UMAP projection (postUMAP_1 and postUMAP_2) displayed 119,840 cells from 19 patient samples annotated into 26 distinct cell populations based on comprehensive marker gene expression profiling. The major cell type categories include epithelial populations (epithelial cells, endothelial cells), T-cell subsets (B cells, CD4^+^ T cells, CD8^+^ T cells, CD4^+^ Tem, CD8^+^ Tem, CD8^+^ Tex, CD8^+^ Trm, Treg, proliferating T cells), myeloid lineages (macrophages, monocytes, neutrophils, cDC, and pDC), other immune populations (NK cells, γδ T cells, plasma cells, B cells, and other immune cells), and stromal components (matrix fibroblasts, vascular fibroblasts, and mast cells). Cell populations occupy distinct transcriptomic spaces, reflecting biological and functional specialization within the tumor microenvironment. CD4_Tem indicates CD4^+^ T-effector memory cells; CD4_Tex, CD4^+^ T-exhausted cells; CD8_Tem, CD8+ T-effector memory cells; CD8_Tex, CD8^+^ T-exhausted cells; CD8_Trm, CD8^+^ T-resident memory cells; cDC, conventional dendritic cells; gd T-cell, γδ T cells; pDC, plasmacytoid dendritic cells; Treg, regulatory T cells (CD4^+^, CD25^+^, FOXP3^+^).

Chemokine expression was systematically extracted and quantified from integrated scRNA-seq data. For each identified cell type, average expression levels were calculated for 17 chemokines (CXCL5, CXCL6, CXCL8, CXCL9, CXCL10, CXCL11, CXCL12, CXCL16, CCL2, CCL3, CCL4, CCL5, CCL7, CCL8, CCL20, CCL27, and CCL28) and 5 chemokine receptors (CXCR2, CXCR3, CCR1, CCR2, and CCR5) using Seurat AverageExpression function applied to log-normalized data. Percent expression (percentage of cells expressing each gene above the detection threshold) was computed for each cell type, and dot plots, heat maps, and bar graphs were generated.

## RESULTS

Analysis of scRNA-seq data across 119,840 quality-checked cells from 19 patient samples in 3 BTC transcriptomic data sets (GSE210066, GSE201425, and GSE213452) revealed that different immune cell types are present inside the BTC TME, such as B cells, CD4 T cells, CD4^+^ T-effector memory cells, CD8^+^ T-effector memory cells, CD8^+^ T-exhausted cells, CD8^+^ resident memory cells, regulatory T cells, γδ T cells, conventional dendritic cells, plasmacytoid dendritic cells, macrophages, mast cells, monocytes, neutrophils, NK cells, plasma cells, proliferating T cells, and other types of T cells. In addition to immune cells, epithelial cells (including malignant BTC cells), endothelial cells, matrix fibroblasts, vascular fibroblasts, and other unassigned cell types were also identified by analyzing the scRNA-seq data (Fig. 2).

Chemokine expression across the 3 BTC transcriptomic data sets (GSE210066, GSE201425, and GSE213452) revealed variable chemokine profiles within the BTC TME. A comprehensive assessment of 17 chemokine ligands identified variable expression patterns across individual samples, with mean log_2_-transformed expression values ranging from 0 to 2 (Fig. 3).

**FIGURE 3:**
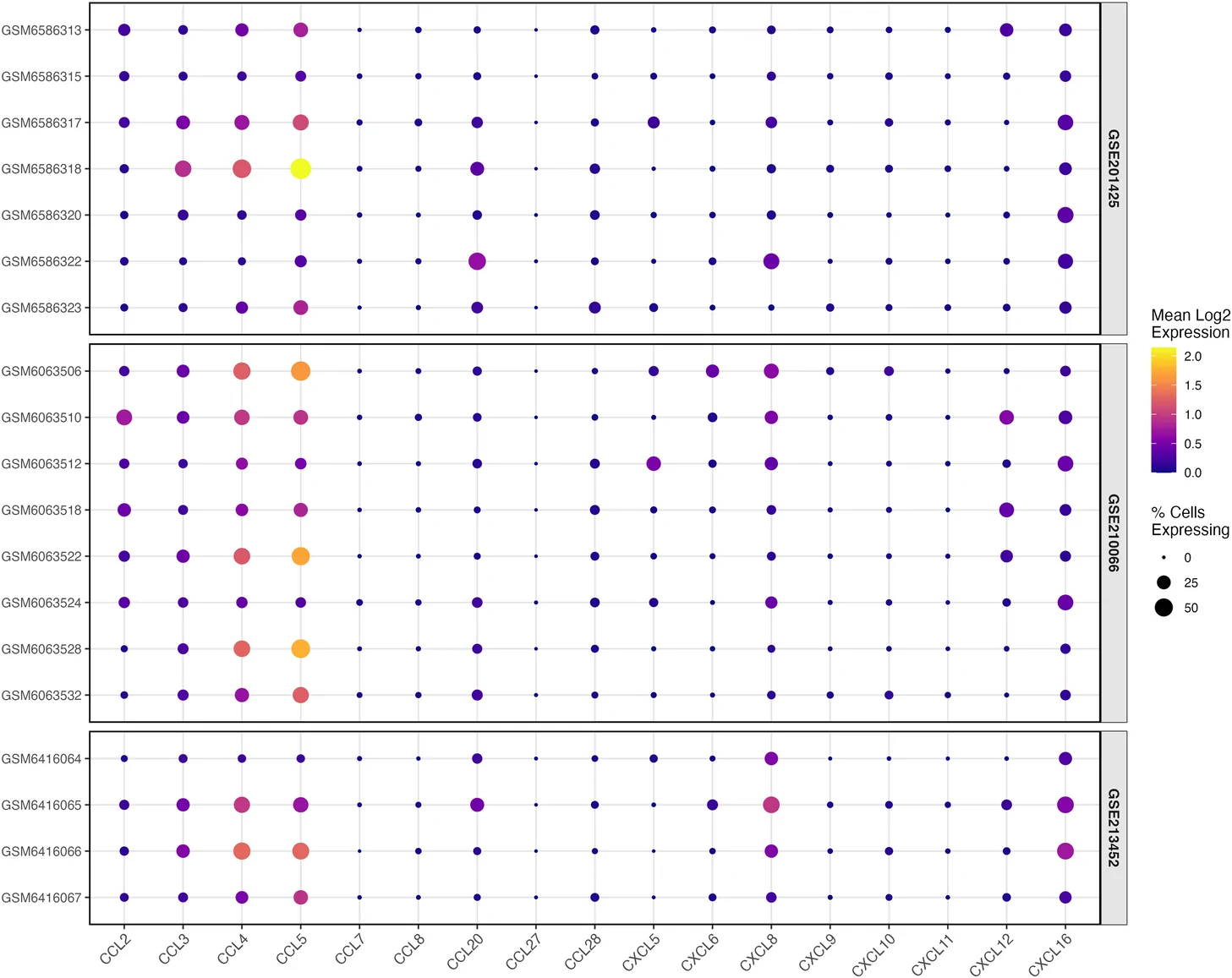
Chemokine expression profile across BTC samples. Chemokine expression in samples from 19 BTC patients. Dot plot visualization stratified by data set origin showing mean log_2_-transformed expression (color intensity; scale: 0 to 2.0) and percentage of cells expressing each chemokine (dot size; 0%, 25%, 50% of cells) for 17 chemokine genes (CCL2, CCL3, CCL4, CCL5, CCL7, CCL8, CCL20, CCL27, CCL28, CXCL5, CXCL6, CXCL8, CXCL9, CXCL10, CXCL11, CXCL12, and CXCL16) quantified across all 119,840 integrated cells. CD4_Tem indicates CD4^+^ T-effector memory cells; CD4_Tex, CD4^+^ T-exhausted cells; CD8_Tem, CD8^+^ T-effector memory cells; CD8_Tex, CD8^+^ T-exhausted cells; CD8_Trm, CD8^+^ T-resident memory cells; cDC, conventional dendritic cells; gd T-cell, γδ T cells; pDC, plasmacytoid dendritic cells; Treg, regulatory T cells (CD4^+^, CD25^+^, FOXP3^+^).

From the scRNA-seq data analysis, it is evident across all data sets that there was high expression of CCL4 and CCL5 chemokine ligands in the tumor microenvironment, whereas CCL2 and CCL3 were also expressed variably across the 3 data sets. CCL20 was also variably expressed in different samples across the 3 data sets, whereas the expression of CCL7, CCL8, CXCL9, CXCL10, and CXCL11 was very limited across all 3 BTC data sets (Fig. 3). Across all 3 data sets, CCL5 was expressed in ∼30% of cells. CXCL16 and CCL4 were expressed on average in 24% and 23% of the cells, respectively. CXCL8, CCL3, and CCL20 were expressed in 13% to 15% of cells present in the BTC TME. CCL2, CXCL12, and CCL28 were expressed by only 6% to 10% of cells present in the TME, whereas the expression of CXCL6, CXCL5, CXCL10, CXCL9, CCL8, CXCL11, CCL7, and CCL27 was very limited (<5% of cells) (Fig. 4).

**FIGURE 4:**
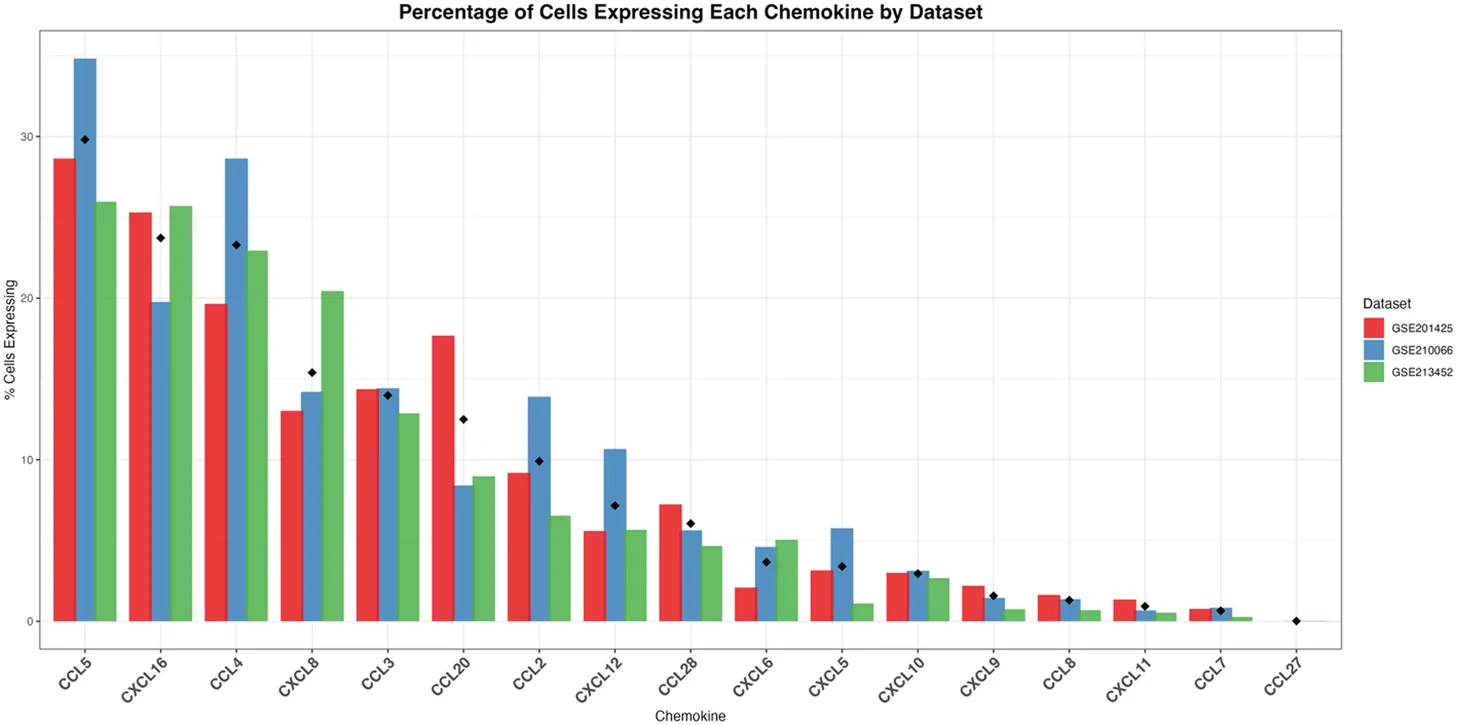
Comparative chemokine cell expression across BTC data sets. Comparative prevalence of chemokine expression across 3 integrated BTC data sets. Grouped bar chart showing the percentage of cells expressing each chemokine (y-axis; 0% to 35% of total cells) stratified by data set origin: GSE201425 (red; n=7 samples), GSE210066 (blue; n=8 samples), and GSE213452 (green; n=4 samples). Black diamond markers indicate the mean expression prevalence across all 3 data sets, enabling rapid visual identification of data set-consistent versus data set-discordant chemokine signatures.

Analysis of single-cell RNA sequencing data (Figs. 5, 6) also showed that CCL5 production or expression was mostly abundant in NK cells, γδ T cells, proliferating T cells, CD8^+^ T-effector memory cells, CD8^+^ resident memory cells, and CD8^+^ exhausted T cells. Similarly, CCL4 is also produced by NK cells, γδ T cells, and various CD8^+^ T-lymphocytes, whereas CCL3 is expressed on macrophages, NK cells, and CD8^+^ exhausted T cells. CCL2 expression signals mainly originate from matrix fibroblasts, vascular fibroblasts, macrophages, and some endothelial cells, which also express CCL2. CXCL16 expression was observed in macrophages and, to some extent, in epithelial cells. Strong expression of CXCL8 was derived from neutrophils and macrophages, and low expression was noted in epithelial cells. Low- to moderate-expression of CCL20 was also noted in epithelial cells as well as in CD4^+^ T-effector memory cells, regulatory T cells, and CD4^+^ exhausted T cells.

**FIGURE 5:**
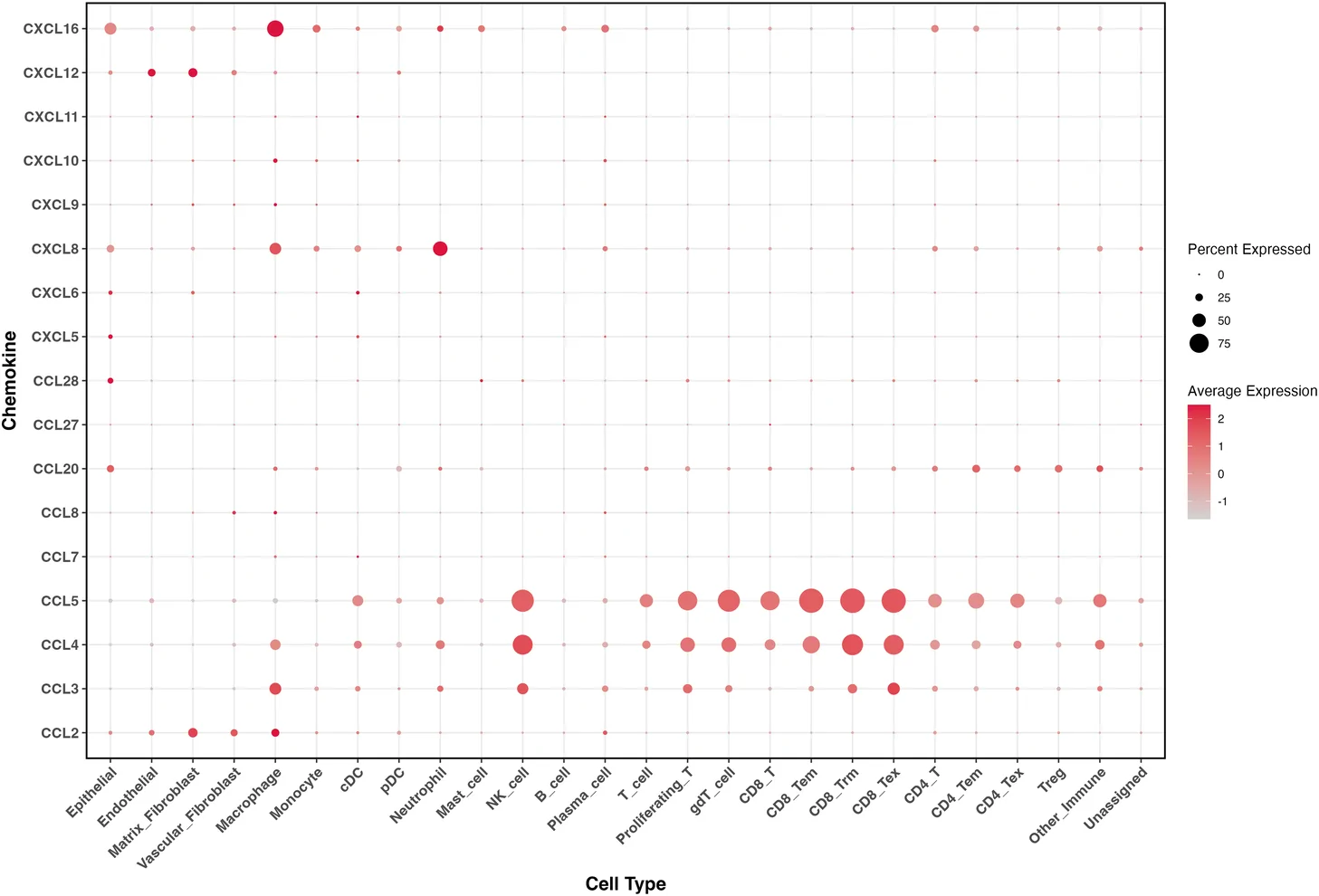
Cell-type-specific chemokine expression in the BTC tumor microenvironment. Chemokine expression profiling across 26 annotated cell types; dot plots displaying mean log_2_-transformed expression (color intensity; scale −1 to 2, red coloration indicating higher expression) and percentage of cells expressing each chemokine (bubble size: 0%, 25%, 50%, and 75% of cells per population) for 17 chemokine genes (CCL2, CCL3, CCL4, CCL5, CCL7, CCL8, CCL20, CCL27, CCL28, CXCL5, CXCL6, CXCL8, CXCL9, CXCL10, CXCL11, CXCL12, and CXCL16) quantified across 119,840 cells from 19 patient samples. CD4_Tem indicates CD4^+^ T-effector memory cells; CD4_Tex, CD4^+^ T-exhausted cells; CD8_Tem, CD8^+^ T-effector memory cells; CD8_Tex, CD8^+^ T-exhausted cells; CD8_Trm, CD8^+^ T-resident memory cells; cDC, conventional dendritic cells; gd T-cell, γδ T cells; pDC, plasmacytoid dendritic cells; Treg, regulatory T cells (CD4^+^, CD25^+^, FOXP3^+^).

**FIGURE 6:**
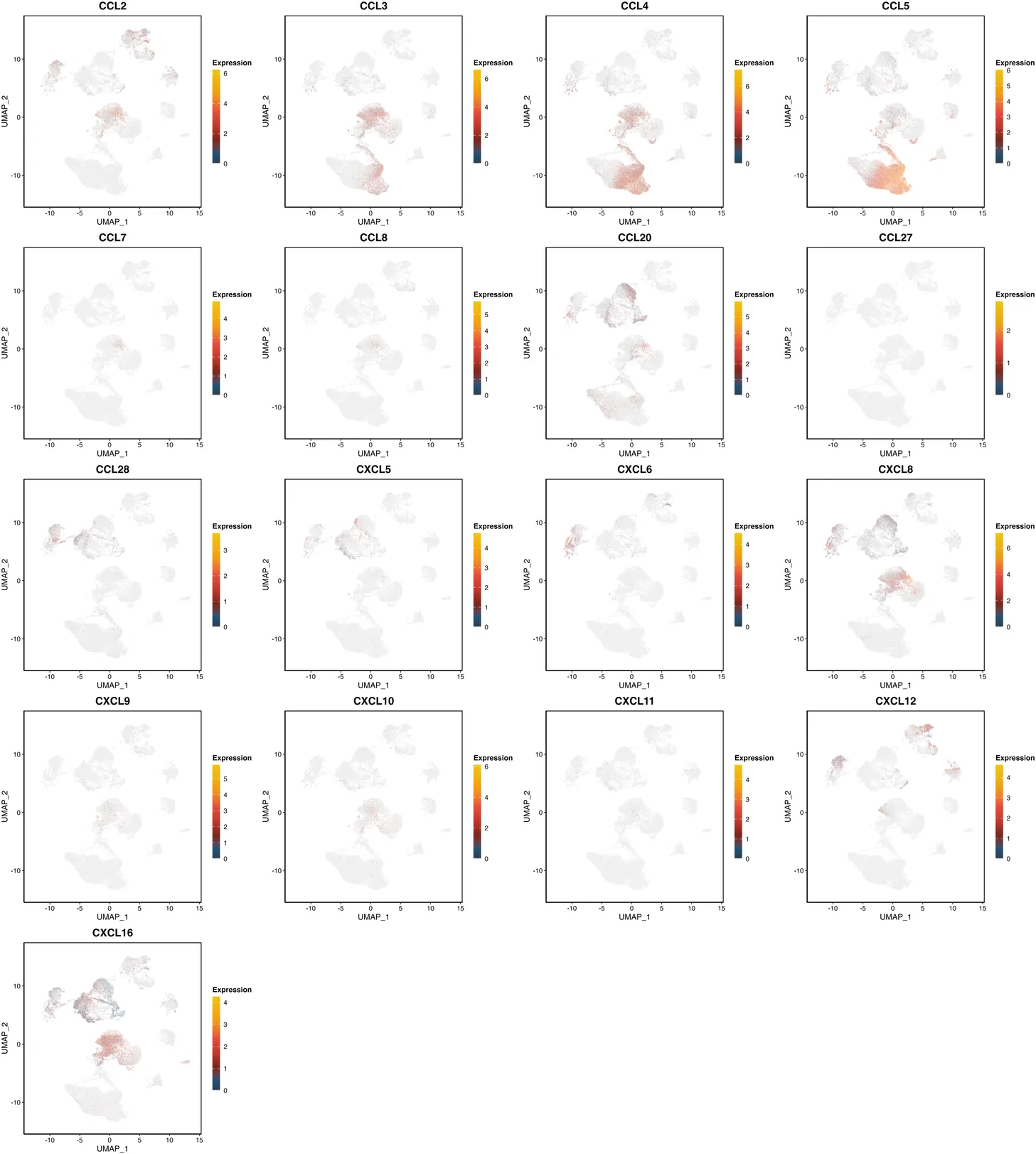
Spatial distribution of chemokine expression across the integrated BTC TME. Feature maps displaying log_2_-normalized expression of 17 chemokine genes overlaid on post-Harmony UMAP embeddings of 119,840 integrated BTC TME cells. Individual UMAP projections (postUMAP_1 × postUMAP_2) are colored by gene expression intensity (blue [low] to yellow [high]; viridis color scale), enabling spatial visualization of chemokine expression patterns across transcriptomic neighborhoods and cell populations.

## DISCUSSION

### Predominance of CCL4, CCL5, and CCL2 in the BTC Microenvironment

Our findings demonstrate a selective chemokine ligand environment within the BTC microenvironment, which presents both opportunities and constraints for γδ T-cell–based adoptive immunotherapy. The predominance of CCL4, CCL5, and CCL2 (Fig. 1) expression aligns with the known chemokine ligands recognized by CCR5^+^ and CCR2^+^ γδ T-cell subsets, particularly Vγ9Vδ2 and Vδ1 T cells, respectively.^15,16^

CCL2 is expressed at mild-to-moderate levels within the BTC TME, as demonstrated by both log_2_ expression (Fig. 3) and percentage-positive cell analyses (Fig. 4). On the basis of a literature review, it is mainly produced by tumor-associated macrophages, stromal cells, and endothelial cells in response to inflammatory cues.^25^ In our data set, similar expression signals were observed in fibroblasts and endothelial cells. CCL2 drives the recruitment of CCR2^+^ γδ T-cell subsets, particularly Vδ1 T cells, and drives the infiltration of monocytes and myeloid-derived suppressor cells (MDSCs), influencing immunosuppression in the TME.^25^ The presence of CCL2 facilitates γδ T-cell mobilization, but may simultaneously enhance protumorigenic myeloid cell accumulation, underlining a dual role in modulating immunity and tumor progression in BTC.^26^

In our analysis, CCL4 (also known as macrophage inflammatory protein-1β or MIP-1β) was well expressed in the BTC TME in most of the samples, and abundance was noted on NK cells, γδ T cells, and various T-lymphocytes. CCL4 is a key chemokine involved in immune cell recruitment and is relevant to γδ T-cell biology, particularly in the context of TME and inflammatory responses, through interaction with receptors such as CCR5.^27^ Within the TME, CCL4 is primarily produced by infiltrating immune cells, including activated macrophages, effector T cells (both CD4^+^ and CD8^+^), NK cells, and dendritic cells, along with fibroblasts and tumor-associated endothelial cells that respond to inflammatory cytokines such as TNF-α and interferon-γ (IFN-γ).^28^

In our analysis, CCL5 (RANTES) showed the highest expression in most types of infiltrating immune cells. Usually, CCL5 is produced by multiple cell types within the TME, including macrophages, dendritic cells, and activated T cells, and has historically been associated with the enhanced recruitment of proinflammatory effector lymphocytes. The high expression of CCL4 and CCL5 suggests a fundamental capacity for CCR5-mediated chemotaxis of Vγ9Vδ2 T cells expressing this receptor. However, the biological significance is mitigated by evidence that CCL5 simultaneously recruits tumor-promoting cell populations, including regulatory T cells (Tregs) and tumor-associated macrophages (TAMs).^29,30^ The recruitment of Tregs by CCL5-CCR5 signaling creates an immunosuppressive microenvironment that can limit effector T-cell function and tumor control, potentially counterbalancing any beneficial γδ T-cell infiltration.

### CCL3: an Additional Chemotactic Axis Supporting γδ T-Cell Mobilization

In the scRNA-seq analysis, moderate expression of CCL3 (macrophage inflammatory protein-1α [MIP-1α]) was observed across BTC cohorts, and the expression signal was mostly from macrophages, NK cells, and CD8^+^ exhausted T cells, representing a substantial cellular population capable of establishing chemotactic gradients. CCL3 has been identified to be produced by diverse cell types in the TME, including activated macrophages, dendritic cells, effector T cells, NK cells, mast cells, endothelial cells, and fibroblasts.^31^ Its expression is increased by inflammatory cytokines (TNF-α, IL-1β, and IL-6) and TME inflammatory signaling. γδ T cells themselves also generate CCL3 upon TCR activation, establishing a positive feedback loop that amplifies γδ T-cell recruitment and activation in response to tumor antigens.^31^

### Receptor Expression and Relevance for γδ T-Cell Recruitment

CCL3 functions primarily through its interaction with CCR5 (the shared receptor for CCL4 and CCL5),^18^ with secondary activity through CCR1.^15^ A significant subset of γδ T cells expresses CCR5, indicating that CCL3 complements and reinforces the chemotactic gradients established by CCL4 and CCL5. This multiligand engagement of CCR5 creates a redundant and reinforced chemotactic system; tumors expressing CCL3, CCL4, and CCL5 simultaneously establish potent CCR5-mediated recruitment axes. In addition, certain γδ T-cell subsets express CCR1, an alternative receptor for CCL3.^15^

### The Paradox of CXCL8 Expression

Bimodal expression of CXCL8 is particularly noteworthy from a therapeutic perspective. While CXCL8 represents a ligand for CXCR1^+^ Vδ1 T-cell recruitment, the chemokine simultaneously promotes tumor angiogenesis, neutrophil recruitment, and differentiation into protumorigenic MDSCs.^32,33^ In samples demonstrating high CXCL8 (GSM6063506, GSM6416065, GSM6586322), the chemokine environment may paradoxically favor myeloid skewing over γδ T-cell infiltration, particularly if CXCR1^+^ Vδ1 frequencies are limited to the peripheral blood of individual patients. Emerging data regarding CXCL8-mediated recruitment of neutrophils and MDSCs emphasize that high CXCL8 expression within the TME may create an immunosuppressive landscape dominated by myeloid populations with enhanced protumorigenic functions.^34,35^

### CXCL9 and CXCL10 Deficiency and Implications for CXCR3^+^ T-Cell Recruitment

A therapeutically concerning finding is the near-absent expression of CXCL9 and CXCL10 (interferon-γ-inducible protein 10 [IP-10]) in the BTC TME. Notably, minimal CXCL10 expression contrasts with chemokine patterns in inflammatory cancers (such as melanoma or colorectal carcinoma), where IFN-γ-driven chemokine expression is characteristic.^36^ Recent studies have demonstrated that tumor-intrinsic epigenetic mechanisms, particularly polycomb repressive complex 2 (PRC2)-mediated histone H3 lysine-27 trimethylation (H3K27me3) modification, can silence Th1-type (attracts T-helper type 1 lymphocytes) chemokines, including CXCL9 and CXCL10, thereby limiting effector T-cell trafficking into the TME and enabling tumor immune escape.^37^

### Epithelial-Homing Chemokines: CXCL16 Expression Supports Epithelial γδ T-Cell Mobilization

In contrast to the near-complete absence of CCL20 (except for GSM6586322 and GSM6416065), our analysis revealed that CXCL16 demonstrated mild-to-moderate expression across the BTC TME. CXCL16, the ligand for CXCR6, is recognized as a mediator of epithelial tissue homing and is particularly enriched in intraepithelial γδ T cells that provide barrier immunity within mucosal surfaces and the gastrointestinal epithelium.^38,39^ These tissue-resident γδ T cells represent a distinct developmental and functional lineage, distinct from circulating Vγ9Vδ2 T cells, and specialize in epithelial immune surveillance.

### The CXCL12-CXCR4 Axis and Immune Suppression

In a few samples, such as GSM6063510 and GSM6063518, mild-to-moderate expression of CXCL12 was noted. CXCL12 binds to CXCR4 receptors expressed on Vδ1 and Vδ2 subtypes of γδ T cells, and CXCR4 expression on γδ T cells is enhanced under specific inflammatory conditions (eg, HIV infection).^40^ It is noteworthy that the CXCR4-CXCL12 axis is well-established as a key driver of CXCR4^+^ MDSC, Tregs, and tumor-associated macrophage (TAM) infiltration into the TME, promoting angiogenesis and facilitating cancer progression.^41^

### Therapeutic Opportunity: Selective Enrichment of CXCR6^+^ γδ T Cells

BTC specifically arises from the biliary epithelium. The mild-to-moderate CXCL16 expression suggests a rational therapeutic opportunity: selective expansion and enrichment of CXCR6^+^ γδ T cells would be preferentially recruited to BTC tumors,^42^ where they could establish epithelial immune surveillance functions specific to biliary tract malignancy. Moreover, CXCR6^+^ γδ T cells frequently express additional markers associated with tissue residency and epithelial trafficking (including CCR9, integrin α4β7, and tissue-type lymphocyte homing receptors), enabling sustained accumulation within epithelial compartments.

### Integrated Analysis: Multiaxis Chemokine-Driven Recruitment Framework

Our comprehensive analysis revealed that despite its fundamentally immunosuppressive phenotype, the BTC TME establishes multiple reinforced chemokine-driven infiltration axes supporting γδ T-cell recruitment.

Primary recruitment axes involving CCL5-CCR5, CCL4-CCR5, and CCL2-CCR2 signaling establish gradients capable of supporting Vγ9Vδ2 and Vδ1 T-cell infiltration. These are the dominant chemokine systems in the BTC microenvironment.

Secondary recruitment axes involving CCL3-CCR5 and CCL3-CCR1 signaling reinforce primary CCR5-mediated infiltration and provide alternative CCR1-dependent pathways for subsets of γδ T cells. This multiligand engagement creates redundant chemotactic signaling that is resistant to selective immune evasion through single-ligand suppression.

Epithelial-homing axes involving CXCL16-CXCR6 signaling, although more modest in magnitude, establish epithelial-homing gradients supporting intraepithelial γδ T-cell localization and barrier function.

Suppressed recruitment axes involving CXCL9/CXCL10-CXCR3 signaling represent a deficit, reflecting the epigenetic or transcriptional suppression of IFN-γ-responsive immune programs.

This multiaxis architecture suggests that comprehensive γδ T-cell–based adoptive immunotherapy might strategically exploit multiple chemokine systems rather than rely on a single infiltration pathway. The presence of redundant CCR5 ligands (CCL3, CCL4, and CCL5) suggests that CCR5^+^ γδ T cells would encounter highly permissive infiltration.

### Therapeutic Strategies to Enhance γδ T-Cell Recruitment and Function

#### Strategy 1: Multiaxis Chemokine Receptor-Matched Cell Expansion and Engineering

Current clinical-grade expansion protocols utilize zoledronate (a bisphosphonate) and IL-2 to generate large numbers of Vγ9Vδ2 T cells. These expanded cells express high levels of CCR5 and CXCR3^43^ and variable expression of other chemokine receptors, depending on the differentiation state and cytokine milieu during expansion. Selective expansion of CCR5^+^ Vγ9Vδ2, CCR2^+^ Vδ1, CCR1^+^, and CXCR6^+^ epithelial-homing γδ T cells matched the BTC chemokine landscape. The 3-ligand CCR5 engagement strategy (CCL3, CCL4, and CCL5) provides redundancy and resistance to selective immune evasion. Receptor engineering by downregulating CXCR3 and upregulating CXCR6 and tissue homing markers enhances infiltration while minimizing off-target recruitment.

#### Strategy 2: Chemokine Supplementation

Systemic CXCL10 administration rescues IFN-γ responsive recruitment;^13^ CXCL16 upregulation supports epithelial homing. CCL3 supplementation reinforces multiligand CCR5 engagement.^44^

#### Strategy 3: Selective Chemokine Axis Antagonism

CCL2-CCR2 blockade reduces protumorigenic monocyte recruitment while preserving CCR5 ligand-mediated γδ T-cell entry.^45^ CXCR1/CXCR2 antagonism or CXCL8 neutralization attenuates myeloid recruitment in CXCL8-replete tumors, which favors immune cell infiltration; it may also favor γδ T cells.^46^

#### Strategy 4: Multicompartmental Epithelial Immune Surveillance

Cotransfer of CXCR6^+^ epithelial-homing, CCR5^+^ stromal, and CCR2^+^ γδ T cells enables simultaneous infiltration of epithelial and stromal compartments, establishing comprehensive antitumor coverage through multiaxis chemokine targeting.

### Patient-Specific TME Profiling and Heterogeneity

Finally, the striking heterogeneity in chemokine expression across individual patient samples (with 4 to 18 fold variations in mean log_2_ expression between samples) emphasizes the importance of patient-specific TME profiling. BTC represents a biologically heterogeneous disease, and our data reveal that distinct patient tumors express divergent chemokine landscapes, with substantial variations in dominant chemokines, even between samples within the same GEO data set. Therapeutic strategies optimized for 1 patient’s TME may prove suboptimal or entirely ineffective for another. This emphasizes the need for pretreatment chemokine profiling by single-cell transcriptomics, spatial transcriptomics, or multiplexed immunofluorescence to stratify patients and select appropriate γδ T-cell subsets for adoptive transfer.

### Limitations

Although Harmony batch correction mitigates technical bias, transcriptomic profiling cannot confirm protein-level chemokine secretion, γδ T-cell receptor expression, or therapeutic responsiveness. Gene expression data reflect transcriptional signatures without addressing chemokine gradient kinetics or spatial protein distributions. Validation via multiplex immunohistochemistry, flow cytometry, and functional assays is essential to translate transcriptomic findings into therapeutic translation.

## CONCLUSIONS

Our comprehensive analysis of 3 independent single-cell transcriptomic data sets demonstrated that the BTC TME contained a distinctive and multiaxis-selective chemokine profile. The moderate-to-high expression of CCL5, CCL4, CCL3, and CCL2 across patient cohorts establishes a fundamental capacity for the recruitment of CCR5^+^ Vγ9Vδ2, CCR2^+^ Vδ1, and CCR1^+^ γδ T cells, representing a mechanistic foundation for γδ T-cell–based adoptive immunotherapy. Uniquely, the presence of 3 CCR5 ligands (CCL3, CCL4, and CCL5) creates a redundant and reinforced chemotactic architecture resistant to selective immune evasion mechanisms. However, deficiencies in CXCL9 and CXCL10 (reflecting the suppression of IFN-γ-driven immune responses) represent barriers to broader γδ T-cell infiltration. Importantly, mild-to-moderate CXCL16 expression indicates that the BTC microenvironment retains the capacity to support epithelial γδ T-cell recruitment through CXCL16-CXCR6 signaling. The same chemokine axes that support γδ T-cell accumulation—namely CCL2-CCR2, CXCL8-CXCR1, and CXCL12-CXCR4 signaling—also serve as recruitment signals for immunosuppressive populations, including MDSCs, Tregs, and TAMs. This chemokine-driven recruitment paradox may present a barrier to therapeutic efficacy.

The heterogeneity in chemokine expression between individual patient samples emphasizes that only a subgroup of BTC patients might benefit from γδ T-cell adoptive immunotherapy. Patient stratification based on pretreatment chemokine profiling, combined with rationally designed γδ T-cell engineering (including selective enrichment of CCR5^+^, CCR2^+^, CCR1^+^, and CXCR6^+^ populations), selective chemokine supplementation, and targeted antagonism of immunosuppressive chemokine axes, represents a promising approach to overcome the immunosuppressive BTC microenvironment.
